# Phylogenetic classification of natural product biosynthetic gene clusters based on regulatory mechanisms

**DOI:** 10.3389/fmicb.2023.1290473

**Published:** 2023-11-08

**Authors:** Alberto C. Rodriguez-Sanchez, Luz A. Gónzalez-Salazar, Lorena Rodriguez-Orduña, Ándres Cumsille, Agustina Undabarrena, Beatriz Camara, Nelly Sélem-Mojica, Cuauhtemoc Licona-Cassani

**Affiliations:** ^1^Centro de Biotecnologia FEMSA, Escuela de Ingeniería y Ciencias, Tecnológico de Monterrey, Monterrey, Mexico; ^2^Centro de Biotecnología Daniel Alkalay, Universidad Técnica Federico Santa María, Valparaíso, Chile; ^3^The Novo Nordisk Foundation Center for Biosustainability, Technical University of Denmark, Lyngby, Denmark; ^4^Centro de Ciencias Matemáticas, UNAM, Morelia, Mexico; ^5^Integrative Biology Unit, The Institute for Obesity Research, Tecnológico de Monterrey, Monterrey, Mexico

**Keywords:** gene regulation, biosynthetic gene cluster, natural product, transcription factor, histidine kinase, two component system

## Abstract

The natural products (NPs) biosynthetic gene clusters (BGCs) represent the adapting biochemical toolkit for microorganisms to thrive different microenvironments. Despite their high diversity, particularly at the genomic level, detecting them in a shake-flask is challenging and remains the primary obstacle limiting our access to valuable chemicals. Studying the molecular mechanisms that regulate BGC expression is crucial to design of artificial conditions that derive on their expression. Here, we propose a phylogenetic analysis of regulatory elements linked to biosynthesis gene clusters, to classify BGCs to regulatory mechanisms based on protein domain information. We utilized Hidden Markov Models from the Pfam database to retrieve regulatory elements, such as histidine kinases and transcription factors, from BGCs in the MIBiG database, focusing on actinobacterial strains from three distinct environments: oligotrophic basins, rainforests, and marine environments. Despite the environmental variations, our isolated microorganisms share similar regulatory mechanisms, suggesting the potential to activate new BGCs using activators known to affect previously characterized BGCs.

## Key message

Our study represents the first approach that classifies biosynthetic gene clusters according to a regulatory structure. Through the study of known activators of well-characterized BGCs, we have identified common patterns in regulatory mechanisms, offering potential activators for previously unexplored BGCs. This research contributes to NP discovery by presenting a framework for identifying potential activators for novel BGCs, opening the door to the discovery of new Natural Products.

## 1. Introduction

The exploration of natural products (NPs) from microbial sources has played a key role in the search of pharmaceuticals, insecticides, and herbicides (Katz and Baltz, 2016). However, discovery of novel chemical classes of antimicrobials has dramatically decreased since the discovery of lincosamides in 1963 (González-Zorn and Escudero, 2012; Aminov, 2017). To address this challenge, extensive sequencing efforts have been dedicated to expanding the genomic space of biosynthetic gene clusters (BGCs) within microbial genomes. Through bioinformatics and extensive curation, different databases have rapidly emerged categorizing NPs according to chemical structures, functions and other features (Hammami et al., 2010; Diminic et al., 2013; Ichikawa et al., 2013; Tremblay et al., 2016; Palaniappan et al., 2020; Blin et al., 2021). The presence of BGCs in bacteria has a taxonomic correlation across strains of the same species (Adamek et al., 2018). However, horizontal gene transference also plays an important role in BGC acquisition by evolutionary pressure (Osbourn, 2010).

The goal of harnessing the full potential of NPs often centers around understanding the molecular mechanisms governing their biosynthesis. Notably, NP production is subject to regulation influenced by several factors, including pleiotropic and pathway-specific factors. This regulation often involves a complex interplay of regulatory genes (transcription factors and sigma factors), within the BGCs (Ortet et al., 2012; Otani et al., 2022). While biosynthetic gene-centered databases like antiSMASH (Blin et al., 2019), MIBiG (Kautsar et al., 2020) or Bactibase (Hammami et al., 2010) have been crucial for studying the genetic diversity, databases focusing on accessory genes (e.g., regulatory genes) are less utilized. Furthermore, despite the success of accessory genes-centered databases in studying regulation [such as ARTS (Mungan et al., 2020) and P2TF (Ortet et al., 2012; Otani et al., 2022)], limited information is available regarding regulatory genes across the diversity of BGCs.

Regulatory mechanisms are crucial for controlling the transient expression of NPs in nature and artificial cultures (Barka et al., 2016). Genes on bacteria are regulated by a series of sensing-effector mechanisms, the sensing mechanism consists of a sensor domain (input domain) responsible for detecting a specific stimulus, and the effector mechanism consists of a domain responsible for making the corresponding action in response to the stimuli (output domain). The regulatory mechanism in bacteria is directed by Transcription Factors (TFs), which regulate genes by activating their output domain in response to a stimulus. One-component systems (OCS), the main regulatory mechanism found in microbial BGCs, are TF with both sensing and effector domains (Ulrich et al., 2005). On the other hand, external stimuli are sensed by Two-component systems (TCSs), which consist of an autophosphorable histidine kinase (HK) and a Response Regulator (RR) (McLean et al., 2019). Typical HKs have an extracellular sensor domain, a conserved histidine residue domain, and a catalytic domain. RRs have a phosphoacceptor region with a conserved aspartate residue and an effector domain (DNA interaction, proteins interaction, or enzymatic region) (Groisman, 2016; McLean et al., 2019). There have been some analyses of regulatory genes of BGC in *Streptomyces* and other relevant NP producers. For example, McLean et al. (2019) described a survey of potential TCS on *S. venezuelae* and some of them show a regulatory function on antibiotics. Also, a pangenome analysis recently reported that *Streptomyces* Antibiotic Regulatory Proteins (SARPs) TFs are less conserved on *Streptomyces* species (strain-specific) and control the BGCs in which they are encoded (Otani et al., 2022).

Accessing microbial NPs from environmental strains remains challenging because replicating natural conditions under artificial settings is practically impossible. Given the conservation of BGCs among taxonomic-related strains, known regulatory mechanisms can be transposed to environmental strains to activate un-expressed (silent) BGCs (Martínez-Burgo et al., 2019; Krause et al., 2020). While, several studies now incorporate ecological insights into the design of drug discovery pipelines (Som et al., 2017), novel approaches are required to understand potential BGC activation mechanisms and facilitate NP discovery from environmental strains.

In this study, we propose a framework to identify closely related regulatory elements from environmental strains from the Cuatro Cienegas Basin (CCB), the Calakmul rainforest, in Mexico; and the Comau fjord, Penas Gulf, and Valparaíso beach in Chile. By utilizing Hidden Markov Models from the Pfam database to detect key protein domains and a well curated BGC database (MIBiG), we generated a database of 2,694 BGCs including 784 BGCs from our strain collection. We conducted phylogenetic analyses of regulatory mechanisms (TFs and TCS) on both known and predicted BGCs to study activation mechanisms. This research contributes to our understanding of the diversity of regulatory mechanisms within BGCs and enhances our ability to predict activators in environmental isolates, thereby advancing the field of NP discovery.

## 2. Materials and methods

### 2.1. Genome sequences

High quality genomic information from a total of 40 environmental strains are included in this study (Supplementary material). 23 Environmental strains were isolated from Cuatro Cienegas Basin, and eight strains from Calakmul in Mexico. Genome sequences were obtained using methods previously reported (Gallegos-Lopez et al., 2020). On the other hand, nine genomes of strains isolated from various locations in Chile (4 from Valparaiso, 4 from Comau Fjord, Huinay and 1 from Penas Gulf) were also included. All sequences are available on GenBank database, and raw data were deposited to a BioProject with accession numbers provided in Supplementary material. All genomes were assembled using PATRIC (Davis et al., 2020) and annotated using Rapid Annotation of microbial genomes using Subsystems Technology (RAST) (Overbeek et al., 2014). Preliminary taxonomic classification was determined based on average amino acid identity (AAI) using The Microbial Genomes Atlas (MiGA) (Rodriguez-R et al., 2018).

### 2.2. BGC detection

Biosynthetic gene cluster was predicted in all genomes using antiSMASH version 6.0 (Blin et al., 2021). BGC completeness was calculated using the BiG-FAM database (Kautsar et al., 2021). Only regulatory proteins of complete BGCs were used in the analysis. Sequences of BGC reference were downloaded from Minimum Information about a biosynthetic gene cluster (MIBiG) (15).

### 2.3. Histidine kinase detection

Histidine kinase class I and II have a different structure, class I has a dimerization and histidine phosphoacceptor (DHp) domain and class II has a histidine-containing phosphotransfer (HPt) domain, but both share a similar CAtalytic domain (CA domain). To detect all HK first all CA-containing were recovered and classified as class I (DHp-containing) and class II (HPt-containing). Proteins with Histidine Kinase CA domain, a domain present in HK class I and II, were used to recover all potential HK in MIBiG and genome sequences. Protein sequences with CA domain were detected with HMMER version 3.3.2 (Nov 2020)^1^ using Hidden Markov models (HMM) of CA domains (Table 1) from Pfam-A database at default values (per-sequence *E*-value of 0.01 or less and a per-domain conditional *E*-value of 0.01 or less).

**TABLE 1:** CA domains selected from the Pfam database.

Pfam accession	Family	Summary
PF02518	HATPase_c	Histidine kinase-, DNA gyrase B-, and HSP90-like ATPase
PF13581	HATPase_c_2	Histidine kinase-like ATPase domain
PF13589	HATPase_c_3	Histidine kinase-, DNA gyrase B-, and HSP90-like ATPase
PF13749	HATPase_c_4	Putative ATP-dependent DNA helicase recG C-terminal
PF14501	HATPase_c_5	GHKL domain

Histidine phospho-acceptor domain (DHp) was detected in CA domain proteins. DHp was detected with HMMER using HMM from the Pfam database (Table 2) at default values. Class II HK were protein sequences with a CA domain and Histidin phosphotransfer domain (Hpt), and Class I HK were protein sequences with a CA domain and DHp. HKs from TCS were considered kinases with a Response Regulator next to in the same direction frame.

**TABLE 2:** Histidine phospho-acceptor domains selected from the Pfam database.

Pfam accession	Family	Summary
PF00512	HisKA	His Kinase A (phospho-acceptor) domain
PF01627	Hpt	Histidine phosphotransfer domain
PF02895	H-kinase_dim	Signal transducing histidine kinase, homodimeric domain
PF07536	HWE_HK	HWE histidine kinase
PF07568	HisKA_2	Histidine kinase
PF07730	HisKA_3	Histidine kinase
PF10090	HPTransfase	Histidine phosphotransferase C-terminal domain
PF19191	HEF_HK	Dimerization and histidine phosphotransfer (DHp) domain

### 2.4. Transcription factor and response regulation detection

TFs from BGC were detected using selected TF domains using HMM from the Pfam database (Table 3). TF domains were detected using HMMER with default parameters. RRs were detected from previously detected TF. Response regulator receiver domain (PF0072) with the phosphorable aspartate residue. Were detected and filtered, after that, a TF was classified as Response Regulator if its gen is next to a previously detected HK in the same strand, the rest TF were discarded as RR. All TF with both, the OmpR domain (PF00486) and Bacterial Transcriptional Activator Domain (BTAD) (PF03704) were classified SARP.

**TABLE 3:** Selected transcription factor in Pfam database.

Pfam accession	Family	Summary
PF00027	cNMP_binding	Cyclic nucleotide-binding domain
PF00158	Sigma54_activat	Sigma-54 interaction domain
PF00165	HTH_AraC	Bacterial regulatory helix-turn-helix proteins, AraC family
PF00196	luxR_family	Bacterial regulatory proteins, luxR family
PF00325	Crp	Bacterial regulatory proteins, crp family
PF00376	MerR	MerR family regulatory protein
PF00440	TetR_N	Bacterial regulatory proteins, tetR family
PF00486	Trans_reg_C_OmpR	Transcriptional regulatory protein, C terminal
PF08664	YcbB	YcbB domain
PF02954	HTH_8_Fis	Bacterial regulatory protein, Fis family
PF03704	BTAD	Bacterial transcriptional activator domain
PF04397	LytTR	LytTr DNA-binding domain
PF08279	HTH_11	HTH domain
PF08769	Spo0A_C	Sporulation initiation factor Spo0A C terminal

### 2.5. Phylogenetic analysis

Protein sequences with the same phosphoacceptor domain or transcription factor domain were used to calculate the phylogenetic tree. All sequences were aligned using MUSCLE (Edgar, 2004) using default parameters. Phylogenetic trees were calculated using FastTree (Price et al., 2010) at default parameters (Jukes-Cantor + CAT model). Microreact (Argimón et al., 2016) web was used for tree visualization. All the phylogenetic trees obtained in this study are available as Supplementary material.

## 3. Results

Our database comprises a total of 2,694 complete BGCs, with 1,910 originating from MIBiG and 784 newly reported from environmental samples in our strain collection. Of these BGCs, 204 contain at least one histidine kinase, while 1,291 encode at least one transcription factor. Notably, 2,213 BGCs do not contain either an HK or TF. We identified a total of 225 HKs, with 137 previously identified in MIBiG BGCs and 88 from environmental samples. Additionally, we identified a diverse array of 2,777 distinct TFs, encompassing 1,952 from MIBiG BGCs and 825 from environmental samples. The remaining 2,213 BGCs do not encode any HKs or TFs within the cluster.

**FIGURE 1:**
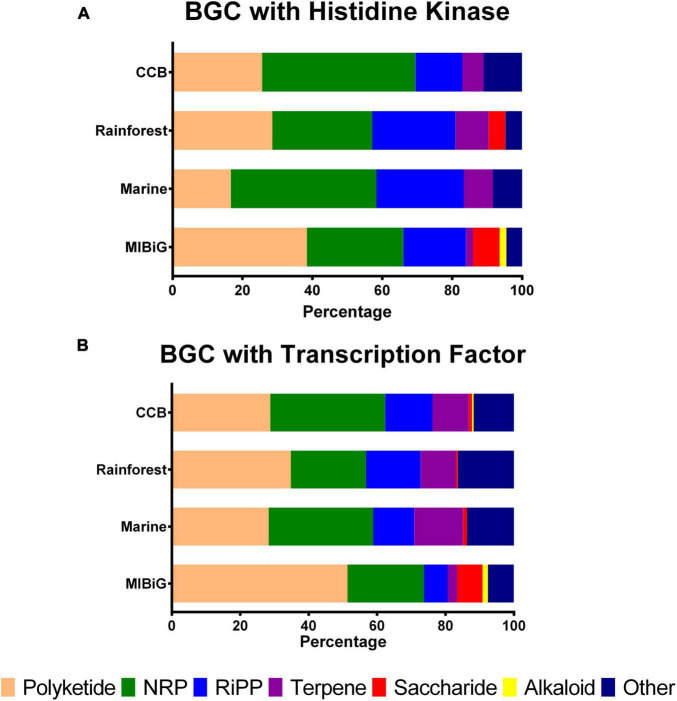
Biosynthetic gene clusters classes distribution with at least one regulatory element by environment. **(A)** BGCs with a histidine kinase by product class **(B)** BGCs with transcription factors by product class. NRPs, non-ribosomal peptide, RiPP, ribosomally synthesized and post-transcriptionally modified peptides.

To analyze the preference for NP classes among actinobacterial strains from different environments, we examined the distribution of BGC classes. We calculated the percentage of BGC classes that contain at least one HK, as shown in Figure 1A. For BGCs belonging to strains from the Cuatro Cienegas Basin that contain an HK, we observed the following distribution: Polyketide (25.6%), non-ribosomal peptide (NRP, 43.9%), ribosomally synthesized and post-translationally modified peptide (RiPP, 13.4%), terpene (6.1%) and other (11%). For rainforest samples, the distribution was as follows: polyketide (28.6%), NRP (28.6%), RiPP (23.8%), terpene (9.5%), saccharides (4.8%), alkaloids (0%), and other (4.8%). In contrast, the BGCs from marine strains exhibited the following distribution: polyketide (16.7%), NRP (41.7%), RiPP (25%), terpene (8.3%) and other (8.3%). Finally, the distribution of BGCs from the MIBiG dataset was as follows: polyketide (38.5%), NRP (27.6%), RiPP (17.9%), terpene (1.9%), saccharides (7.7%), alkaloid (1.9%), and other (4.5%).

Histidine kinases were predominantly associated with BGCs from Polyketides in the MIBiG database and rainforest samples, while BGCs from NRP were primarily linked to the Cuatro Cienegas Basin and marine strains. Alkaloid-containing BGCs were exclusively found in the MIBiG dataset, and saccharide-containing BGCs were identified in both Cuatro Cienegas Basin and MIBiG samples.

On the other hand, Figure 1B illustrates the percentage of BGC classes containing TFs. In BGCs from the Cuatro Cienegas Basin with TFs, we observed the following distribution: polyketide, 28.8%; NRP, 33.6%; RiPP, 13.8%; terpene, 10.6%; saccharide, 0.9%; alkaloid, 0.5% and other, 11.8%. For rainforest BGCs with TFs, the distribution was as follows: polyketide, 34.8%; NRP, 22%; RiPP, 16%; terpene, 10.2%; saccharide, 0.6%; and other, 16.4%. Conversely, BGCs from marine environments with TFs exhibited the following distribution: polyketide, 28.3%; NRP, 30.6%; RiPP, 12%; terpene, 14.3%; saccharide, 1.1% and other, 13.7%. Lastly, BGCs from the MIBiG dataset with TFs were distributed as follows: polyketide, 51.4%; NRP, 22.4%; RiPP, 6.9%; terpene, 2.8%; saccharide, 7.5%; alkaloid, 1.5% and other, 7.6%.

Similar to the distribution of HKs, BGCs from the polyketide class were more prevalent in strains from the rainforest and the MIBiG dataset, with polyketides accounting for over half of the BGCs containing TFs in the MIBiG dataset. Similarly, NRPs with TFs were more common in both the Cuatro Cienegas Basin and marine strains. Notably, BGCs containing TFs from the alkaloid class were absent in rainforest and marine strains.

**FIGURE 2:**
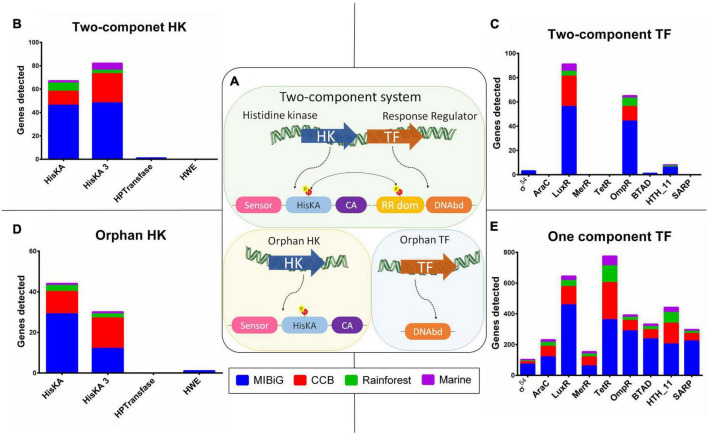
Histidine kinase and transcription factor Pfam domain distribution. **(A)** Schematic representation two-component system (TCS), structure of HK without a conjugated response regulator (orphan HK), and one-component system TF (OCS TF). **(B)** HK with a response regulator from a TCS. **(C)** TF with DNAbd, and response regulator domain (RR dom) from a TCS. **(D)** HK without response regulator (orphan HK). **(E)** TF with a DNA binding domain (DNAbd), from OCS.

We identified the presence of Two-Component system regulatory mechanisms when both a HK and a TF were encoded in tandem, transcribed within the same strand, and the TF encoded a response regulator protein domain (PF00072). Additionally, we classified HKs without a corresponding TF as “orphan HKs,” and TFs without an associated HK as “orphan TFs.” All regulatory proteins, including HKs and TFs, were categorized based on their regulatory mechanisms into TCS, orphan HKs, or orphan TFs, as depicted in Figure 2A. Our analysis revealed that 66% of the HKs are part of a TCS, with a relative abundance of 69% in the MIBiG dataset, 58% in BGCs from strains in the Cuatro Cienegas Basin, 66% in BGCs from the rainforest, and 80% in BGCs from strains originating from marine environments.

Within the TCS, HisKA and HisKA3 were the predominant contributors among HKs, accounting for 49.3 and 49.7%, respectively (Figure 2B). In contrast, HPTransfase and HWE made minimal contributions, comprising only 1 and 0%, respectively. Among RR, LuxR and OmpR protein family domains were the major contributors, representing 54 and 38%, respectively. BTAD and Helix-turn-helix (HTH_11) protein families contributed to RRs with 0.6 and 4.7%, respectively. Sigma-54 domain was present in 2% of all RRs, while the remaining domains were not associated with a TCS (Figure 2C). As expected, the MIBiG database significantly contributed to the presence of TCS due to its higher number of BGCs compared to environmental samples. The LuxR domain was predominant in RRs across most cases, with occurrences in 50.9% of MIBiG RRs, 67.6% of CCB RRs, and 66.7% of marine RRs. The exception was in rainforest RRs, where the LuxR domain was found in 58.3% of cases.

In a similar pattern to HKs within the TCS, we observed that HisKA and HisKA3 domains were present in almost all orphan HKs, accounting for 58.7 and 40%, respectively, while HWE contributed 1.3% (Figure 2D). Among the TFs associated with the orphan control system (OCS) mechanism, the TerR family dominated with a representation of 23%. The LuxR domain constituted the second most prevalent family at 19.1% within OCS mechanisms, followed by the OmpR family at 11.6%. Notably, TetR was more prevalent in OCS from the Cuatro Cienegas Basin, rainforest, and marine environments, accounting for 29.7, 34, and 29.6%, respectively (Figure 2E). In contrast, within the MIBiG dataset, 22.7% of OCS-associated BGCs contained a LuxR domain.

**FIGURE 3:**
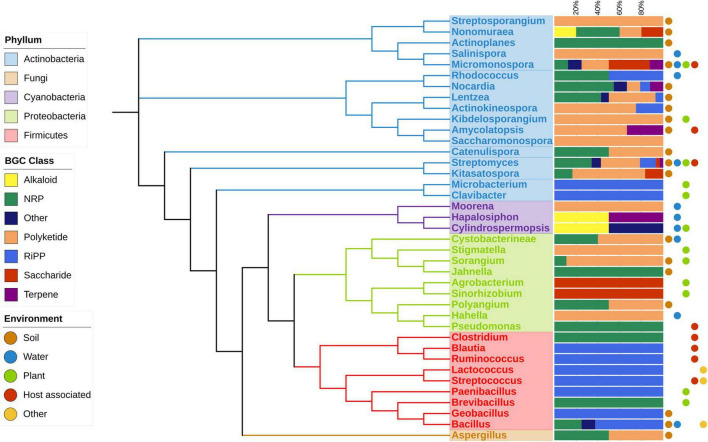
Phylogenetic distribution of strains with BGCs with a HK TCS, BGC class and isolation environment are depicted. The phylogenetic tree was inferred using rRNA of the most representative species on each genus. Dots at the right of the figure show the type of environments where at least one strain was isolated.

To examine the impact of environmental factors on BGC diversity across different genera, we analyzed the diversity of BGC classes and compared them to the isolation environments of each genus. Our analysis revealed a clear relationship between the occurrence and diversity of BGCs and the variety of environments where microorganisms were isolated. Figure 3 illustrates the diversity of BGC classes containing a HK from a TCS across orders and phyla. Host-associated environments encompass both pathogenic and symbiotic cases. Some phyla exhibit limited diversity; for instance, the Firmicutes phylum contains only NRP, RiPP, and other compound classes. Proteobacteria exhibit NRP, Polyketides, and Saccharides. In contrast, the Actinobacteria phylum boasts the highest number of members, covering all BGC classes. Within this phylum, the *Micromonospora* and *Streptomyces* genera are found in all environments, while most orders in the database were isolated from only one or two environments, with *Bacillus* being an exception, isolated from three different environments.

### 3.1. Distribution of His kinase A domain

**FIGURE 4:**
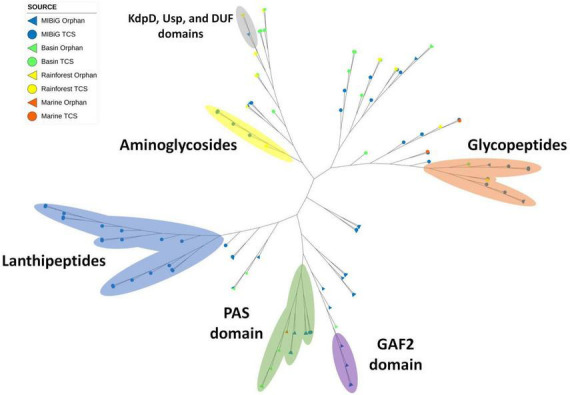
Phylogenetic tree of histidine kinase with HisKA domain (PF00512). Regions depicted in blue, orange, and yellow correspond to clades with HK from lanthipeptides, glycopeptides, and aminoglycosides producing BGCs, respectively. Regions in green, purple, and gray correspond to proteins with PAS, GAF2, and KdpD-Usp-DUF domains, respectively.

Histidine kinase A (PF00512) and the catalytic ATP-binding domain (PF02518) were identified in a total of 111 protein sequences, with 75 originating from the MIBiG database and 36 from environmental samples. Specifically, HKs associated with lanthipeptide-producing BGCs formed a distinct clade within the phylogenetic tree (Figure 4, blue region), all originating from Gram-positive bacteria. In contrast, glycopeptide-producing BGCs form a clade (Figure 4, orange region) and were primarily composed of actinobacterial strains, including two environmental strains: one from *Lentzea* sp. in the Cuatro Cienegas Basin and another from *Streptomyces* sp. in the rainforest.

A smaller clade comprised of aminoglycoside-producing bacteria was also identified, with one *Streptomyces* sp. strain from a rainforest sample clustering within it (Figure 4, yellow region). Diverse protein domains were detected within the potential sensor region. Notably, an atypical structure containing an osmosensitive K + channel His kinase sensor domain: Kdp (PF02702), universal stress protein family (PF00582), and domain of unknown function DUF4118 (PF13493) was identified within a *Streptomyces*-exclusive clade. This clade included three *Streptomyces* spp. from rainforest sequences and one laidlomycin-producing BGC from *Streptomyces* sp. CS684 in the MIBiG database (Figure 4, gray region).

Additionally, the PAS domain was detected in 20 sequences, with 12 of them forming a distinct clade consisting of diverse bacterial genera associated with toxin and stress-response BGCs (Figure 4, green region). This clade encompassed a wide range of microorganism genera, including *Pseudomonas*, *Agrobacterium*, *Cylindrospermopsis polyangium*, and *Jahnella*. Furthermore, four environmental samples from *Nocardia* sp. and one from *Streptomyces* sp. were found within this clade, originating from the Cuatro Cienegas Basin and marine environments, respectively. Finally, GAF2 domains were identified in four sequences from soil bacteria, including two from *Sorangium cellulosum*, one from *Streptomyces* sp. CNT302, and one from *Amycolatopsis tolypomycina* (Figure 4, purple region).

### 3.2. Distribution of histidine kinase 3

**FIGURE 5:**
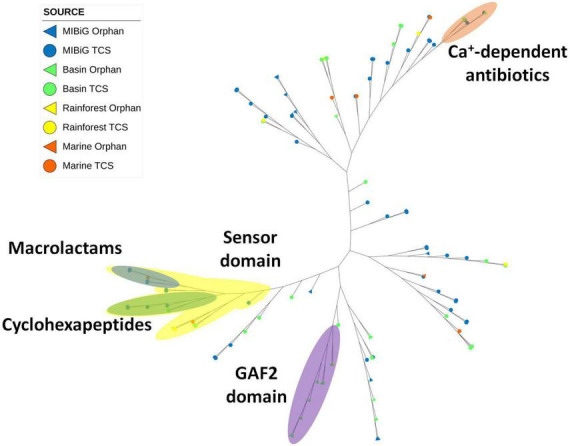
Phylogenetic tree of histidine kinase HisKA_3 domain (PF07730). The orange region corresponds to HK from calcium-dependent antibiotics BGC. The yellow region corresponds to sequences with the sensor domain (PF13796). The blue region corresponds to sequences from macrolactams BGC. The green region corresponds to cyclohexapeptides BGC. The purple region corresponds to HK with the GAF2 domain (PF13185).

Histidine Kinase 3 (PF07730) and the catalytic ATP-binding domain (PF02518) were identified in a total of 112 sequences, including 60 from MIBiG, 40 from the Cuatro Cienegas Basin, 7 from marine environments, and 5 from the rainforest. A distinct clade associated with calcium-dependent antibiotics BGCs was identified (Figure 5, orange region). Within this clade, two environmental samples were clustered, one originating from *Streptomyces* sp. SS52 and the other from *Lentzea* sp. CC55. Notably, this clade predominantly comprised soil actinobacteria or samples obtained from soil environments.

Another clade with BGCs responsible for producing macrolactams was identified (Figure 5, blue region). This clade primarily consisted of actinobacterial strains isolated from aquatic environments, with one sample originating from the marine environment. Another clade associated with the production of cyclohexapeptides was also observed. This clade comprised *Streptomyces* sp. strains from MIBiG and was a subclade of a larger group containing the sensor domain (PF13796) in the sensor region. This sensor domain is associated with proteins containing histidine kinase 3 and the catalytic ATP-binding domain. Notably, the majority of these histidine kinases were associated with BGCs responsible for producing NRPs products.

Additionally, a clade containing environmental samples characterized by the presence of the GAF2 domain in the N-terminal region was identified (Figure 5, purple region). All members within this clade were isolated from soil environments, with only one from the rainforest, while the rest were from the Cuatro Cienegas Basin. Most of the BGCs within this clade produced products classified as polyketides.

### 3.3. Distribution of LuxR-family domains

**FIGURE 6:**
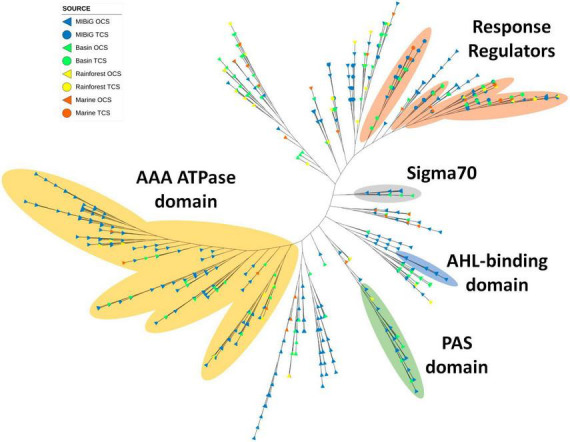
Phylogenetic tree of TF with LuxR domain family (PF00196). Yellow region corresponds to TF clade with AAA ATPase domain. Regions depicted in orange, gray, blue, and green correspond to TFs with response regulator protein domain (PF00072), sigma70, N-acyl homoserine lactone (AHL) binding domain and PAS domain, respectively.

A total of 735 transcription factors carrying the LuxR domain were identified across all databases, with 513 originating from MIBiG, 143 from the Cuatro Ciénegas Basin, 44 from the rainforest, and 35 from marine environments. Notably, polyketide and NRPs classes were the most abundant, constituting 35.8 and 36.2% of the total, respectively. Furthermore, the AAA ATPase domain (PF13191) was detected in 191 sequences featuring a LuxR domain. Most of these sequences clustered within a distinct clade (Figure 6, yellow region). This domain is involved in various cellular functions, all of which require energy derived from ATP. These functions encompass roles such as transcriptional activation, and 36 sequences within this clade are derived from environmental samples.

Four closely situated clades primarily corresponded to LuxR proteins with response regulator activity. Approximately half of the response regulators were associated with NRPs metabolites, followed by polyketides (32.5%). Interestingly, response regulators were evenly distributed across these clades. Another small clade featuring the sigma 70 region was detected, with members predominantly originating from the Cuatro Ciénegas Basin and all corresponding to *Streptomyces* strains. The transcription factors associated with BGCs in this clade were responsible for the production of BGCs and polyketides, except for those linked to saccharide (kanamycin) and terpene (tiancilactone) products.

Additionally, a clade contained LuxR proteins with the autoinducer binding domain, with all sequences originating from the MIBiG database and associated with polyketide and NR-like BGCs. Among these BGCs, four belonged to *Pseudomonas*, four to *Burkholderia*, and one to *Methylobacter*. Finally, the PAS domain was identified in 27 sequences clustered within a single clade, with six originating from environmental samples: five from the Cuatro Ciénegas Basin and one from the marine environment. Most of the transcription factors within this clade were associated with polyketide BGCs.

### 3.4. TetR family diversity

The TetR domain emerged as the most prevalent domain among transcription factor proteins associated with BGCs, with 22% of all transcription factors featuring this domain. Following closely was the LuxR domain, found in 21% of all transcription factors. Notably, 40% of these transcription factors were linked to polyketide BGCs, while 33% were associated with NRPs. Furthermore, 46% of all transcription factors were sourced from the MIBiG database.

**FIGURE 7:**
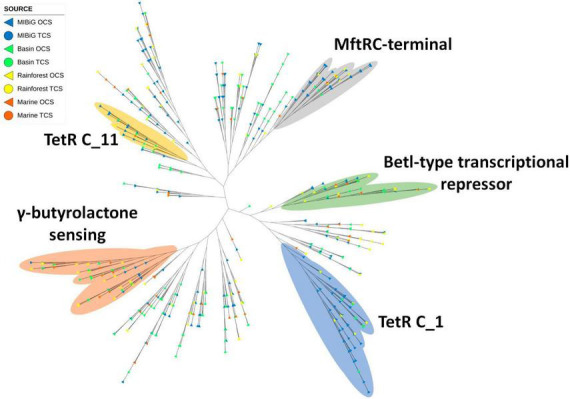
Phylogenetic tree of TF with a TetR family domain (PF00440). Yellow and blue regions correspond to TF clades with a TetR C_1 and TetRC_11 domain (PF02909 and PF16859) respectively. Regions depicted in gray, green, and orange correspond to TF with MftRC-terminal, Betl-type transcriptional repressor domain, and γ-butyrolactone sensing domain, respectively.

One clade comprising 42 proteins featured the TetR C-terminal 14 (MftR C-terminal domain) (Figure 7). The majority of these transcription factors were associated with polyketides, with 14 originating from environmental samples. Among the environmental samples, rainforest sequences were more prevalent, accounting for six transcription factors. Another 37 transcription factors were identified with the TetR C-terminal 11 domain, with 12 of these sequences originating from environmental samples. Within the MIBiG database, polyketides were more common, while NRPs were more prevalent among environmental samples.

A separate clade containing 40 transcription factors featured the TetR C-terminal 6 domain. This clade included a higher proportion of environmental samples (23 proteins). Similar to the TetR C-terminal 11 clade, sequences from the MIBiG database were predominantly associated with polyketides, while environmental samples showed a preference for NRPs. Additionally, a clade encompassing 66 proteins contained the TetR C-terminal 1 domain. Among all TetR C-terminal proteins, this domain was the most prevalent. Within this clade, polyketide and NRP BGCs were more common, with 22 and 20 BGCs, respectively. Lastly, from a review of the literature, a clade of transcription factors activated by γ-butyrolactone was identified. This clade comprised five proteins (ScbR, ScbR2, SabR, CprA, and CprB) clustered together.

### 3.5. OmpR family

**FIGURE 8:**
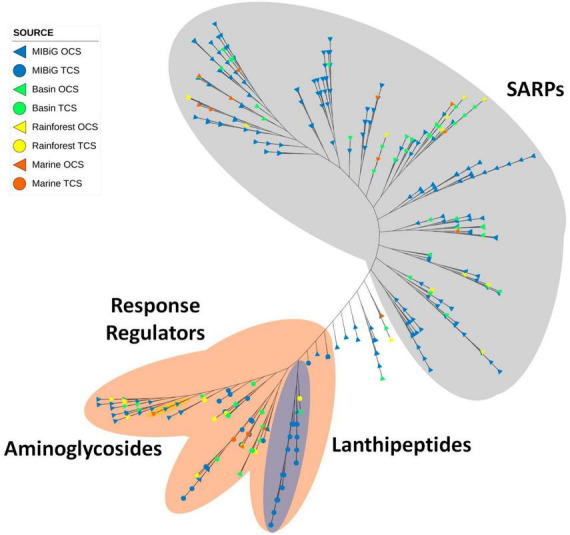
Phylogenetic tree of TF with OmpR family domain. Gray area corresponds to proteins with SARP structure. The orange area corresponds to proteins with RR structure. The blue area indicates a clade with lanthipeptide-produced BGC. The yellow area corresponds to aminoglycoside-produced BGC.

A total of 456 proteins containing the OmpR family domain. Among them, 50.9% are found in polyketie BGCs, and 25.0% are located in NRP-producing BGCs. Phylogenetic trees reveal a clade of proteins with a bacterial transcriptional activator domain. The presence of these two domains is characteristic of SARP. The majority of sequences in this clade originate from *Streptomyces*, accounting for 76% in our database (72% overall). All product classes are evenly distributed along the tree (see Figure 8).

In Figure 8, all 66 RR proteins from TCS were grouped within the orange area. Protein sequences from lanthipeptide-producer BGCs were clustered in the blue area of Figure 8. Within the SARP clade, most sequences from environmental samples are grouped into conserved clades. Specifically, the majority of the 11 samples sequences within SARP were detected in polyketide-producing BGCs, whereas MIBiG sequences in the same clade are also from polyketide-producing BGCs.

Most proteins with a RR-structure belong to a TCS and correspond to NRP-producing BGCs. Within these clades, one subclade with an RR structure does not have a histidine kinase associated with it; most of these are part of polyketide-producing BGCs. Environmental samples are represented in slightly higher proportion among RRs from TCS (33.3%) compared to SARP (25.3%).

### 3.6. Helix-turn-helix family

**FIGURE 9:**
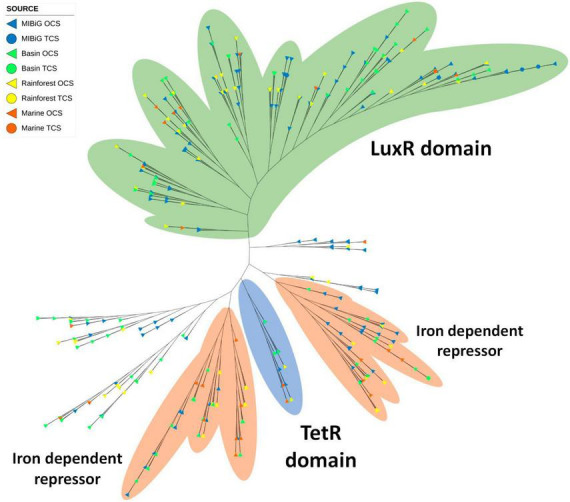
Phylogenetic tree of transcription factor of Helix-turn-helix family (PF08279). The green area corresponds to sequences with an LuxR domain. The orange area corresponds to sequences with Iron dependent repressor domain (PF01325). Blue area contains the sequences with the TetR domain.

A total of 450 TFs encode an HTH domain (PF08279), with the majority originating from NRPs and polyketide-producing BGCs (41.6 and 33.3%, respectively). The distribution by isolation site is similar to that of TetR, where 46% of the proteins come from MIBiG database, 30% from the Cuatro Cienegas Basin, 16% from rainforests, and 8% from marine environments. A significant portion of these proteins also contains a LuxR domain, as shown in the green area in Figure 9. In this clade, NRPs and polyketide BGCs are common, RiPPs at 28, 29, and 24%, respectively. More than half of these proteins originate from the MIBiG database, accounting for 57%.

TetR domains were identified in 40 TFs, and many of them are scattered throughout the phylogenetic tree. Among these 40 sequences, 7 form a distinct clade of TFs with the TetR domain. Most of them are associated with polyketide BGCs, followed by NRPs BGCs. The Iron repressor domain (PF01325) was detected in 125 TFs, forming two closely related clades. These two clades are separated by the TetR clade. Within all TFs containing an Iron repressor domain, 42% are associated with polyketide BGCs, and 38% are linked to NRPs BGCs. Additionally, potential siderophore-producing BGCs from environmental samples were identified, including heterobactin and coelichelin.

## 4. Discussion

The relationships between organisms and their environments can influence genetic variation among species, and this variation is correlated to the environment independently of physical locations (Wang and Bradburd, 2014). To investigate diversity and the correlation between regulatory mechanism variations and the regulation of BGCs in different environments, we conducted a bioinformatic analysis of regulatory elements in 42 strains from three distinct environments: 24 strains from a low-phosphate cambric-like environment (the Cuatro Cienegas Basin), 9 strains from the Calakmul rainforest in Mexico; and 9 strains from various marine environments in Chile. We also included well curated BGCs from MIBiG database. Our analysis primarily focused on the phylogenetic relationships among 2,777 selected transcription factors, which are the primary known mechanisms of gene regulation, 225 histidine kinases (HKs), the primary known mechanism for environmental sensing, and the union of both, which encompassed 168 two-component systems (TCSs). TFs and HK isolated from ES do not show a significant difference compared to the MIBiG database, this can be explained by the size of the sample used, with few organisms from each environment, Tidjani et al. (2019) found that microorganisms isolated from the same environment, same genus, even same sample can have different genes, at a scale of mm3, especially in BGC regions. To find a difference in the environment-exclusive regulation mechanism a big sample should be used.

Our analysis revealed that one-component systems are the primary regulatory system for BGCs. However, a substantial number of BGCs lack a regulatory mechanism within the cluster, suggesting external regulatory mechanisms. Regulatory genes located outside the BGCs, with multiple regulation targets or pleiotropic effects, control many secondary metabolites or morphological development (Lu et al., 2017). Most BGCs with a TF or an HK corresponded to the polyketides or NRPs products. This might be attributed to a bias in natural product research, resulting in an overrepresentation of these two molecular families in databases. In contrast, among other BGC classes, only five saccharide BGCs (three from CCB, one from the rainforest, and one from marine environment) were detected as complete, but only two from CCB had a TF or an HK. Additionally, only one alkaloid BGC from the environmental samples was detected, but it did not have a TF or an HK.

In the HisKA phylogenetic analysis, a clade of lanthipeptide BGCs was observed, with all HKs within this clade being part of a two-component system. For instance, nisin and Bovicin HJ50, two lanthipeptides, are well-known natural products with an autoinducible mechanism (Ni et al., 2011; Özel et al., 2018). Some defined clades containing natural products from similar chemical families were also identified, such as glycopeptides and aminoglycosides. In the glycopeptide clade, various glycopeptides were grouped together, including A40926, A-47934, teicoplanin, UK-68,597, feglymycin, auroramycin, and kistamicin A. Interestingly, three natural products in this clade, namely rapamycin, feglymycin, and auroramycin, are also part of this clade. Two environmental samples producing NRPs (*Lentzea* sp. CC55 from CCB and *Streptomyces* sp. KL110B from the rainforest), were also grouped in this clade. The aminoglycoside clade included gentamicin, sisomicin, and catenulisporolides (a glycosylated macrolide). Additionally, this clade included an environmental sample, *Streptomyces* sp. KL110B from the rainforest, which produces a polyketide.

The HisK 3 domain phylogenetic tree has a marked clade with BGCs for calcium-dependent antibiotics from the NRPs class, which included two of our environmental samples, *Lentzea* sp. CC55 and *Streptomyces* sp. SS52, both from CCB, with one of them having the potential to produce CDA1. Another different clade within the same analysis contained proteins with the putative sensor domain (PF13796), which was identified in proteins with the HisK 3 domain. This clade encompassed two subclades of cyclic proteins: macrolactams and cyclohexapeptides. The macrolactams subclade included BGCs from salinilactam, lobosamide, and sceliphrolactam, and one of the nodes corresponded to an environmental sample, *Streptomyces* sp. Vc67-4 from the marine environment, which has the potential to produce sceliphrolatam. The cyclohexapeptides subclade included BGCs similar to desotamide, curacomycin, dechlorocuracomycin, and ulleungmycin, along with two environmental samples from CCB, *Lentzea* sp. CC55, and *Streptomyces* sp. CC208A. Most of the members of the sensor clade produced cyclic NRPs.

The LuxR domain was the predominant contributor to the response regulator. The LuxR family is associated with quorum sensing and molecular sensing (Santos et al., 2012). Within the LuxR phylogenetic analysis, two major branches were identified. One of these branches contained the AAA ATPase domain, also known as the AAA + superfamily, which is involved in regulatory functions (Snider et al., 2008), including σ54-related transcription factors (Khan et al., 2022). In this branch, none of the TFs were part of a TCS, and it included environmental samples from all environments. Santos et al. (2012) reported an analysis of LuxR proteins in Actinobacteria genomes, revealing that only 0.81% of proteins with a LuxR domain also had an AAA ATPase domain. For the same correlation we found a total of 31.7% proteins. In three different clades on the phylogenetic analysis, we observed that most of the RR from TCS were associated with environmental samples from all environments sampled. Out of the 637 TFs belonging to the actinomyceted, only 13% (83 TFs) were RR, which differed from the 53% previously observed (Santos et al., 2012). In one of the clades, we identified proteins with a sigma70 domain, and four environmental samples, all from different *Nocardia* species from CCB, were included, and they were predicted to produce NRPs.

In addition, a clade containing N-Acyl homoserine lactone (AHL) binding domains was identified from previously characterized Gram negative bacteria. AHL is a known molecule involved in quorum sensing in Gram negative bacteria while GBL molecules are used for Gram positives microorganisms (Santos et al., 2012). We also observed a clade with PAS domains [PAS (PF00989), PAS_4 (PF08448), and PAS_9 (PF13426)]. Interestingly, 3.9% of Actinobacteria TFs were found to encode PAS domains, compared to the 0.10% reported previously (Santos et al., 2012). PAS domains are associated with sensing factors like oxygen, light, and redox states and are widely distributed across living organisms. In our analysis, we detected five *Streptomyces* strains within the PAS clade, four of which were isolated from CCB (with the potential to produce 2-methylisoborneol and candicidin) and one from rainforest (with a 2-methylisoborneol like BGC).

The TetR domain emerged as the most prevalent protein family among TF BGCs, accounting for 21.9% of the cases. The TetR family typically acts as a repressor in most instances (Ramos et al., 2005). Several proteins from our database, including ChlF1, ScbR, ScbR2, SabR, CprA, and CprB, had previously been reported as transcriptional repressors (Li et al., 2016). Some of these proteins are regulated by the presence of γ-butyrolactone, a molecule utilized as a hormone-like signal in quorum sensing, morphological differentiation, and secondary metabolism (Romero-Rodríguez et al., 2015). TetR is generally present in BGCs to repress the production of NPs until specific conditions release the signal. GBL can activate BGCs containing a TetR domain, particularly within the GBL sensing clade observed in the TetR phylogenetic tree.

The TetR phylogenetic tree revealed four marked clades. One of these branches includes the MftR C-terminal domain (PF17754) from environmental samples (rainforest, marine, and CCB). Another branch included TFs with the TetR C-terminal domain 1 (PF02909) and BetI-type transcriptional repressor, C-terminal (PF13977), both of which are associated with antibiotic expression and osmoregulation via pumps (Ramos et al., 2005). The TetR C-terminal 1 clade includes environmental samples from the rainforest and CCB, while the BetI-type clade includes samples from the three environments tested. Another branch featured TFs with the TetR C-terminal domain 11 (PF16859), which is linked to antibiotic expression, multidrug pumps, and toxin response (Ramos et al., 2005). Additionally, one of the branches contained a clade with GBL sensing TFs, which included samples from all environments and featured a predicted BGC for GBL in *Streptomyces* sp. CC219B from CCB.

OmpR domain proteins are typically part of SARP or TCS. Nearly all reported genes containing an OmpR domain positively regulate their BGCs (Guder et al., 2002; Ni et al., 2011; Li et al., 2020), and many of them are essential to produce their respective BGC. A significant portion of reported OmpR-containing proteins with activation mechanisms belongs to SARPs associated with antibiotic-producing BGCs. OmpR is predominantly utilized as part of a SARP or TCS, serving as an activator, which makes them potential tools for enhancing NP production through genetic manipulation (Krause et al., 2020). The OmpR phylogenetic tree featured two main clades, one consisting almost exclusively of SARPs, and the other branch including RRs and OCS. Within this branch, a clade of lanthipeptides was identified, comprising RR from the HisKA HK on the lanthipeptide clade. A small clade containing aminoglycosides was also detected, with one marine isolate.

Most DNA-binding proteins possess a helix-turn-helix motif, which is responsible for DNA binding in almost 95% of transcription factors (Ramos et al., 2005). In our phylogenetic analysis, we identified three major branches. One of these branches comprised TFs with the LuxR domain, previously detected in samples from all environments tested. Within this branch, only seven RRs were present, including two in isolates from the rainforest (with the potential to produce sarpeptin) and the marine environment. Additionally, this branch contained a clade with the Iron-dependent repressor, N-terminal DNA binding domain (PF01325). Another clade consisted of TFs with the previously identified TetR domain, although none of them possessed the Iron-dependent repressor. Isolates from all environments were found within this branch, including *Streptomyces* strains potentially producing venemycin, chloramphenicol, and 7-prenylisatin from CCB and the rainforest. The Iron-dependent repressor was commonly observed in multiple clades of the HTH phylogenetic tree, with four such clades and 27.8% of TFs featuring this domain. When considering a possible relationship between BGCs producing siderophores and the presence of the Iron-dependent repressor, we detected three siderophore BGCs (two for pyoverdine and one for yersiniabactin) from MIBiG, as well as one previously reported potential siderophore BGC (heterobactin) *Rhodococcus* sp. H-CA8f. However, none of these BGCs contained the Iron-dependent repressor. Some Nocardia strains from CCB possessed the Iron-dependent repressor and had the potential to produce a siderophore, although with similarities of 72% and 63% for heterobactin, and 27% for coelibactin.

## 5. Conclusion

In this study, we propose a phylogenetic analysis of regulatory elements linked to biosynthesis gene clusters. Our analysis allowed us to explore the diversity (based on protein domain information) of regulatory mechanisms present in curated BGCs from database. More importantly, our analysis permits to cross-correlate regulatory mechanisms of newly sequenced environmental samples in order to formulate informed hypothesis on activation of BGCs. As a proof of principle, we conducted a comprehensive genome analysis of Actinobacteria isolated from three distinct environments: the low-nutrient Cuatro Ciénegas Basin, the Calakmul Rainforest in Mexico, and the marine environments of Chile. This approach provided valuable insights into the regulatory mechanisms governing biosynthetic gene clusters (BGCs) across diverse ecological niches. Despite variations in isolation sites, our findings revealed striking similarities in the regulatory elements of similar natural products (NPs) compared to those previously documented in the MIBiG database. This innovative approach holds promise for selectively identifying and activating known NPs with analogous activity or structure, albeit with unique characteristics in environmental strains. Furthermore, it offers the potential to uncover potential elicitors for BGCs in strains suitable for NP discovery. However, it’s important to note that this method is most effective for BGCs with internal regulatory elements and may be less efficient for those relying on external activation mechanisms. This research significantly contributes to our understanding of BGC regulatory mechanisms by exploring the roles of transcription factors (TFs) and histidine kinases (HKs) in relation to NP structures and environmental contexts, offering valuable insights for NP mining endeavors.

## Data availability statement

The datasets presented in this study can be found in online repositories. The names of the repository/repositories and accession number(s) can be found in the article/Supplementary material.

## Author contributions

AR-S: Data curation, Formal analysis, Methodology, Writing – original draft, Writing – review and editing. LG-S: Data curation, Formal analysis, Writing – review and editing. LR-O: Data curation, Formal analysis, Writing – review and editing. ÁC: Formal analysis, Writing – review and editing. AU: Data curation, Writing – review and editing. BC: Data curation, Writing – review and editing. NS-M: Conceptualization, Data curation, Formal analysis, Investigation, Methodology, Supervision, Writing – review and editing. CL-C: Conceptualization, Data curation, Formal analysis, Funding acquisition, Investigation, Methodology, Project administration, Resources, Supervision, Validation, Visualization, Writing – original draft, Writing – review and editing.

## Footnotes

1
http://hmmer.org/
