# Supplementary material for: Phylogenetic classification of natural product biosynthetic gene clusters based on regulatory mechanisms
Source: Front Microbiol. 2023 Nov 8;14:1290473. doi: 10.3389/fmicb.2023.1290473 (PMC10663231; doi:10.3389/fmicb.2023.1290473)
Supplement: Supplementary file 1 [file Data_Sheet_1.docx]

Supplementary Material

Analysis of Genetic Regulatory Elements on Biosynthetic Gene Clusters to Predict Natural Product Activators

Alberto Constantino Rodriguez-Sanchez^1^, Luz A. González-Salazar^1^, Lorena Rodriguez-Orduña^1^, Beatriz Cámara^2^, Andrés Cumsille^2^, Agustina Undabarrena^2^, Nelly Sélem-Mojica^3^, Cuauhtémoc Licona-Cassani^1^*

^1^Escuela de Ingeniería y Ciencias, Tecnológico de Monterrey, Monterrey, México

^2^Centro de Biotecnología Daniel Alkalay, Universidad Técnica Federico Santa María, Valparaíso, Chile

^3^Centro de Ciencias Matemáticas, UNAM, México

*** Correspondence:**Cuauhtemoc Licona-Cassani
clicona@tec.mx

**Supplementary information**

**Supplementary Table 1.** List of Environmental Genomes and NCBI access ID

| Name | Source | Isolation site | Accession |
| --- | --- | --- | --- |
| Nocardia sp. CC227C | This study | Cuatro Cienegas | JASITE000000000 |
| Nocardia sp. CC213A | This study | Cuatro Cienegas | JASFXA000000000 |
| Nocardia sp. CC216A | This study | Cuatro Cienegas | JASFXB000000000 |
| Nocardiopsis sp. CC223A | This study | Cuatro Cienegas | JASFDV000000000 |
| Streptomyces sp. CC224E | This study | Cuatro Cienegas | JASITF000000000 |
| Streptomyces sp. CC208B | Gallegos-Lopez et al, 2020 | Cuatro Cienegas | CP045031 |
| Streptomyces sp. CC216B | This study | Cuatro Cienegas | JASCXK000000000 |
| Streptomyces sp. CC224B | This study | Cuatro Cienegas | JASCXL000000000 |
| Streptomyces sp. CC213D | This study | Cuatro Cienegas | JASCXM000000000 |
| Streptomyces sp. CC219A | Gonzalez-Salazar et al, 2022 | Cuatro Cienegas | JAJPUH000000000 |
| Actinokineospora sp. | This study | Cuatro Cienegas | JAJCXD000000000 |
| Streptomyces sp CC208A | This study | Cuatro Cienegas | JASCXN000000000 |
| Streptomyces sp. CC219B | This study | Cuatro Cienegas | JASCXO000000000 |
| Lentzea sp. cc55 | This study | Cuatro Cienegas | JAJCXE000000000 |
| Brevibacterium sp. H-BE7 | Undabarrena et al, 2016 | Comau Fjord, Huinay | SAMN35531454 |
| Corynebacterium alimapuense sp. VA37-3 | Claverias et al, 2019 | Valparaíso | PTJO00000000 |
| Kocuria sp. H-KB5 | Undabarrena et al, 2016 | Comau Fjord, Huinay | PRJNA977705 |
| Rhodococcus sp. HCA8f | Undabarrena et al, 2018 | Comau Fjord, Huinay | CP023720 |
| Streptomyces sp. G11C | Valencia et al, 2021 | Penas Gulf | JABTTT000000000 |
| Streptomyces sp. H-KF8 | Undabarrena et al, 2017 | Comau Fjord, Huinay | LWAB00000000 |
| Streptomyces sp. VB1 | Cumsille et al, 2017 | Valparaíso | SAMN31307266 |
| Streptomyces sp. Vc67-4 | Cumsille et al, 2017 | Valparaíso | PRJNA891055 |
| Streptomyces sp. Vc74B-19 | Valencia et al, 2021 | Valparaíso | JABTTR000000000 |
| Nocardia sp. CC201C | This study | Cuatro Cienegas | JASCXP000000000 |
| Streptomyces sp. CC210A | Gonzalez-Salazar et al, 2022 | Cuatro Cienegas | JAJPUG000000000 |
| Streptomyces sp. CC216C | This study | Cuatro Cienegas | JASCXQ000000000 |
| Streptomyces sp. CC228A | Gonzalez-Salazar et al, 2022 | Cuatro Cienegas | JAJPUI000000000 |
| Streptomyces rochei CC36 | This study | Cuatro Cienegas | JASCXS000000000 |
| Streptomyces rochei CC48 | This study | Cuatro Cienegas | JASCXT000000000 |
| Streptomyces rochei CC71 | This study | Cuatro Cienegas | JASCXU000000000 |
| Streptomyces sp. SS52 | This study | Cuatro Cienegas | JASCXR000000000 |
| Streptomyces cavernau | This study | Cuatro Cienegas | JASCYJ000000000 |
| Streptomyces sp. KL114A | This study | Calakmul | JASERG000000000 |
| Streptomyces sp KL116D | This study | Calakmul | JASERH000000000 |
| Streptomyces sp. KL122B | This study | Calakmul | JASERI000000000 |
| Streptomyces sp. KL111A | This study | Calakmul | JASGLF000000000 |
| Streptomyces sp. KL118A | This study | Calakmul | JASERJ000000000 |
| Streptomyces sp. KL115B | This study | Calakmul | JASERK000000000 |
| Streptomyces sp. KL109B | This study | Calakmul | JASERL000000000 |
| Streptomyces sp. KL110B | This study | Calakmul | JASERM000000000 |

**Supplementary Table 2.** Phylogenetic trees available online

| Class | Pfam | Link |
| --- | --- | --- |
| Histidine kinase | PF00512 | <https://microreact.org/project/58PUeX7PjHbzWmmX57sVRP-hkpf00512> |
|  | PF07730 | <https://microreact.org/project/cmXrmdNyh8D2HKgyykvvkj-hkpf07730> |
|  | PF10090 | <https://microreact.org/project/8r1bDK4NudV7AnuPB9Sg13-hkpf10090> |
|  | HK_Class-I | <https://microreact.org/project/3YMfxEjEn8MPNMUvhCcuwK-hkclass-i> |
| Transcription factors | PF00027 | <https://microreact.org/project/tfccCVZV4c55bteNBDQoo9-tfpf00027> |
|  | PF00158 | <https://microreact.org/project/k7rZgW1YTNroHxaFpe2yq2-tfpf00158> |
|  | PF00165 | <https://microreact.org/project/eSWSUt64T3ihzAiSq8tGFQ-tfpf00165> |
|  | PF00196 | <https://microreact.org/project/xdWBGLNbpwidLK5Xnhp3Gt-tfpf00196> |
|  | PF00325 | <https://microreact.org/project/ivESFgjUdQCFFVj5FSsdW9-tfpf00325> |
|  | PF00376 | <https://microreact.org/project/feSPafsVxfhfwkPT8ndSvi-tfpf00376> |
|  | PF00440 | <https://microreact.org/project/uFLswTm8Bad1YsK3zpNr7P-tfpf00440> |
|  | PF00486 | <https://microreact.org/project/eFFP3zEVtSyhXvrFsipcCw-tfpf00486> |
|  | PF00486+PF03704 | <https://microreact.org/project/o3NuVpJx2pxgJZZUfPeZYA-tfpf00486pf03704> |
|  | PF02954 | <https://microreact.org/project/wHbKvrNtb28iXETtYcqFCT-tfpf02954> |
|  | PF03704 | <https://microreact.org/project/hpS4XxhAKmCdHxywPuDpUa-tfpf03704> |
|  | PF04397 | <https://microreact.org/project/7rBkTVEyDvKXhNsrAjWGCn-tfpf04397> |
|  | PF08279 | <https://microreact.org/project/hcH5MVU5fYA5ftZQHkwTo7-tfpf08279> |
|  | PF08769 | One gen |
